# Angels and Demons: The Effect of Ethical Leadership on Machiavellian Employees’ Work Behaviors

**DOI:** 10.3389/fpsyg.2018.01082

**Published:** 2018-06-28

**Authors:** Frank D. Belschak, Deanne N. Den Hartog, Annebel H. B. De Hoogh

**Affiliations:** Section of Leadership and Management, Amsterdam Business School, University of Amsterdam, Amsterdam, Netherlands

**Keywords:** Machiavellianism, ethical leadership, organizational citizenship behavior, knowledge hiding, emotional manipulation

## Abstract

Machiavellians can be characterized as goal-driven people who are willing to use all possible means to achieve their ends, and employees scoring high on Machiavellianism are thus predisposed to engage in unethical and organizationally undesirable behaviors. We propose that leadership can help to manage such employees in a way that reduces undesirable and increases desirable behaviors. Studies on the effects of leadership styles on Machiavellian employees are scarce. Here we investigate the relationship of ethical leadership with prosocial (helping colleagues or affiliative OCB) and antisocial work behavior (knowledge hiding and emotional manipulation) for employees who are higher or lower in Machiavellianism. The effect of an ethical leadership style on employees predisposed to engage in unethical behaviors has not been investigated so far. In a cross-sectional multi-source survey study among a sample of 159 unique leader–follower dyads, we find interaction effects between leadership and employee Machiavellianism for prosocial and antisocial work behavior. As expected, employee Machiavellianism comes with reduced helping behavior and increased knowledge hiding and emotional manipulation, but only when ethical leadership is low. Under highly ethical leaders, such increases in organizationally undesirable behaviors of Machiavellian employees do not occur. While the cross-sectional design precludes conclusions about the direction of causality, findings of our study suggest to further explore (and from a practical perspective to invest in) ethical leadership as a potential remedy for undesirable behavior of Machiavellian employees.

## Introduction

The psychological literature describes Machiavellians as master manipulators who are willing to use all possible means to achieve their ends (e.g., Wilson et al., 1996; Jones and Paulhus, 2009). Employees scoring high on Machiavellianism (high-Machs) have been consistently found to engage in a plethora of unethical and counterproductive behaviors including lying, theft, sabotage, and bullying in numerous studies (see Dahling et al., 2012). High-Machs might eventually even contribute to the creation of an unethical organizational culture by acting as role models and signaling to others that “anything goes” (e.g., Felps et al., 2006; Pinto et al., 2008). As a consequence, the recommendation of most studies has been to identify and avoid high-Mach employees (e.g., Dahling et al., 2009; Kiazad et al., 2010). However, high-Mach individuals are proficient in deceiving and manipulating their social environment (Davies and Stone, 2003; McIlwain, 2003), thus it might not always be easy to identify Machiavellians in organizations. Also, some authors have noted that high-Machs do not always engage in unethical and counterproductive behaviors; they also show pro-organizational behavior as long as they feel that this is instrumental for achieving their goals (Wilson et al., 1996; Belschak et al., 2015). As Belschak et al. (2015, p. 1935) argue, organizations cannot always avoid having some Machiavellian employees on board, and they suggest to move the focus toward having a better understanding of how to manage high-Mach employees in a way that reduces organizationally undesirable and increases desirable behaviors. Here, we propose that ethical leadership can offer effective ways to do so.

Research on leading Machiavellian employees is hardly available, and the effects of different leadership styles and behaviors on Machiavellian employees have not received much attention to date. The few existing studies focus on the effects of transformational leadership (Belschak et al., 2015), managerial control (Bagozzi et al., 2013), and leader Machiavellianism (Wisse et al., 2015; Belschak et al., 2016). None of these studies explore how to decrease high-Machs’ highly undesirable tendency to engage in unethical behaviors. Somewhat related, Belschak et al. (2015) address how to increase high-Machs’ pro-organizational behavior and show that transformational leaders, who emphasize the importance of new missions and organizational change, are able to stimulate challenging organizational citizenship behavior (OCB) such as making suggestions for change initiatives in high-Mach followers (Belschak et al., 2015). High-Mach employees have a strong goal orientation and instrumental focus (see Christie and Geis, 1970; Jones and Paulhus, 2009) and by emphasizing the importance of change and change-oriented behavior, showing their appreciation of such change initiatives, and empowering employees to make such changes, transformational leaders seem to stimulate high-Machs in particular to engage in such behavior. Yet, this strict goal orientation of high-Machs also implies that such increases in challenging OCB under transformational leaders might not generalize to a wider range of behaviors (e.g., helping colleagues if this is not clearly to their own benefit) and might not reduce unethical work behaviors (e.g., manipulating, cutting corners, or hiding knowledge from others). To stimulate these types of behaviors, we propose that leaders may need to emphasize specifically the importance of employees showing ethical behavior and hence explicitly engage in ethical leadership.

Ethical leaders (i.e., leaders who demonstrate “normatively appropriate conduct through personal actions and interpersonal relationships,” Brown et al., 2005, p. 120) act as role models of ethical behavior, communicate ethical standards, reward ethical behavior, and punish unethical behaviors (see Den Hartog, 2015). Their behavior sends strong signals to their employees that ethical behavior is important and will be rewarded while unethical behavior is undesirable and will be punished. As noted, high-Machs’ strong goal orientation (“doing what it takes to achieve one’s ends”) should make them particularly sensitive to the signals leaders send about what is appreciated, and high-Machs should hence react to ethical leadership with reduced unethical, antisocial work behavior (manipulation and knowledge hiding) and increased ethical, prosocial behavior (helping colleagues or affiliative OCB). Specifically, we hypothesize that compared to low-Mach employees, high-Mach employees show increased affiliative OCB and decreased knowledge hiding and emotional manipulation under highly ethical leaders and, vice versa, they show less affiliative OCB and more knowledge hiding and manipulation when ethical leadership is low. Greenbaum et al. (2017) argued that abusive leaders stimulate manipulative and unethical behavior in Machiavellians. Here, we similarly reason that low ethical leadership may stimulate unethical behavior such as deception and manipulation, whereas high ethical leadership may inhibit such behavior and rather stimulate ethical behavior including helping others in need rather than manipulating and hiding knowledge from them.

Our study adds to both the literature on leadership and on Machiavellianism. In particular, we contribute to the stream of literature investigating the impact of “dark-side” traits like the dark triad (Machiavellianism, psychopathy, and narcissism; Paulhus and Williams, 2002). While the main effects of Mach are well researched, the interactive effects of Mach in leader–follower interactions and the outcomes of these interactions only received attention more recently (e.g., Nevicka et al., 2011; Den Hartog and Belschak, 2012; Belschak et al., 2015; Wisse et al., 2015). We also add to a stream of research in leadership focusing on how to lead specific groups of employees. Based on their traits and values, employees seem to react differently to their leaders (e.g., Ehrhart and Klein, 2001; De Hoogh and Den Hartog, 2009), and here we investigate the role of Machiavellianism on employees’ reactions to ethical leadership. This study contributes specifically to research on ethical leadership and Machiavellianism by showing that ethical leader behavior is suitable for countering antisocial behavioral tendencies in a group of employees (high-Machs) that bears a high risk of engaging in unethical behaviors (e.g., Dahling et al., 2012). Finally, by studying the effect of employee Machiavellianism on their behavioral reactions to ethical leader behaviors, we provide empirical support for scholars who argue that even high-Mach employees do not always engage in unethical behaviors and are also able to show cooperative behavior (e.g., Wilson et al., 1996; Kessler et al., 2010).

### Machiavellianism in Organizations

In the psychological literature (e.g., Christie and Geis, 1970; Jones and Paulhus, 2009), Machiavellianism is defined as a personality trait that refers to “a strategy of social conduct that involves manipulating others for personal gain, often against the other’s self-interest” (Wilson et al., 1996, p. 285). It is regarded as a quantitative trait which implies that all individuals may show manipulative behavior at times, but some may be prone to showing such behavior more often than others. High-Mach individuals are characterized by a specific constellation of characteristics which can be summarized by (a) a strong goal focus and (b) the willingness to use all possible means to achieve their goals.

High-Machs show a *strong goal focus* and stress achievement and winning (Jones and Paulhus, 2009). This goal focus motivates them to use all possible means to achieve their ends (“winning above all”) and ultimately allows high-Machs to show high performance especially if given the opportunity to manipulate and bend rules (Shultz, 1993; Bagozzi et al., 2013), even under conditions of constrained access to resources (Kuyumcu and Dahling, 2014). Supervisors, however, usually evaluate high-Mach employees less positively than low-Machs (Ricks and Fraedrich, 1999). Thus, while the unmitigated use of all means to achieve their ends helps high-Machs to achieve high performance or other goals, it often negatively affects their evaluations by others at the same time, at least in the long run (see Jones and Paulhus, 2009).

High-Machs’ *willingness to deploy antisocial and unethical strategies* can be explained by several mechanisms. First, Machiavellian individuals have a cynical, negative worldview, always expecting the worst from other people (Christie and Geis, 1970). This provides them with a justification for showing unethical behavior, “others would have acted similarly.” Consistently, high-Machs trusted others less in economic situations than low-Machs (Sakalaki et al., 2007). At the same time, high-Machs are emotionally detached from their own actions, allowing them to engage in unethical behaviors without experiencing negative feelings like guilt or remorse (e.g., McHoskey et al., 1998; Wastell and Booth, 2003). The regulatory social function of (self-conscious) negative emotions is thus not equally strongly available to Machiavellians as it is to those low on Mach (Bagozzi et al., 2013, provide a neurological explanation for this deficit). Finally, Machiavellianism comes with a strong self-focus and egoism (Fehr et al., 1992) resulting in a lack of attachment and commitment toward others or the organization (Zettler et al., 2007). Consistently, McLeod and Genereux (2008) note that high-Machs only lie if *they* profit, not if others profit (i.e., no “white lies”).

The mentioned characteristics of Machiavellianism all provide explanations for high-Machs’ low threshold to engage in unethical behaviors, even when being antisocial and (potentially) harming others, and their lack of willingness to engage in behavior that benefits others if it not also clearly benefits them. In line with the arguments above, high-Mach individuals tend to show a number of unethical and counterproductive work behaviors (see Dahling et al., 2009, 2012). For instance, high-Machs are found to lie and deceive others (e.g., Williams et al., 2010), steal (e.g., Harrell and Hartnagel, 1976), defect during bargaining (Gunnthorsdottir et al., 2002), engage in sabotage (e.g., McLeod and Genereux, 2008), and use emotional manipulation (e.g., Austin et al., 2007). Some studies report that they engage in less helping behaviors (Wolfson, 1981; Becker and O’Hair, 2007), while other studies (e.g., Dahling et al., 2009; Bagozzi et al., 2013) report a non-significant relationship of Mach with OCB, which suggests that moderating variables might play a role here.

Studies on Machiavellianism and leadership are scarce. The limited research available on Machiavellian leaders suggests that high-Mach leaders stimulate less positive responses in their followers than low-Mach leaders (e.g., Den Hartog and Belschak, 2012; Belschak et al., 2016). They are also more often perceived as abusive leaders by their followers (Kiazad et al., 2010). Yet, research has also found that high-Mach leaders can be seen as determined and charismatic by followers (Deluga, 2001), and are able to increase employee engagement when showing ethical leader behavior (even though their effect was less strong than when low-Machs engaged in ethical leader behaviors; Den Hartog and Belschak, 2012). This demonstrates high-Mach leaders’ ability to adapt their behavior to the situation despite of being detached from their followers’ interpersonal concerns (Deluga, 2001; Dahling et al., 2009).

Even fewer studies than on Machiavellian leaders have been conducted on leading Machiavellian employees, and thus the effects of different leadership styles on Machiavellian employees have not received much attention to date. Noteworthy exceptions are the studies by Belschak et al. (2015) who have investigated the reactions of Machiavellian employees to transformational leaders, by Belschak et al. (2016) who explored the effects of high-Mach leaders on high- versus low-Mach followers, and by Wisse et al. (2015) who address the role of all three dark triad traits in leaders and followers. Here, we add to this stream of research by testing the effects of ethical leadership on high-Mach versus low-Mach employees’ ethical (affiliative OCB) and unethical work behavior (knowledge hiding and (emotional) manipulation). To our knowledge, research has not yet explored which leadership style might be effective in reducing high-Machs’ highly undesirable tendency to engage in unethical work behaviors.

### Ethical Leadership and Machiavellian Employees

Ethical leadership can be defined as “the demonstration of normatively appropriate conduct through personal actions and interpersonal relationships, and the promotion of such conduct to followers through two-way communication, reinforcement, and decision-making” (Brown et al., 2005, p. 120). Past research on ethical leadership has shown that such leaders foster their followers’ ethical behavior and decrease their unethical behavior (e.g., Brown et al., 2005; Den Hartog and De Hoogh, 2009; Piccolo et al., 2010; Kalshoven et al., 2011b) and has been linked specifically to OCB (e.g., Mayer et al., 2009; Den Hartog and Belschak, 2012).

Ethical leaders are value driven and act in line with their principles (Brown and Treviño, 2006). They stress the importance of fair, moral, and ethical behavior and the avoidance of unethical behavior, and they live up to the values they espouse (Den Hartog, 2015). Ethical leaders act as role models of ethical behavior and stimulate ethical behavior and conduct by rewarding (ethical employee behavior) and punishing (unethical employee behavior) of their followers. They send strong and clear signals to their employees that ethical behavior is desirable and will be noticed and rewarded while unethical behaviors are undesirable and will be punished when detected. In contrast, leaders low on ethical leadership do not signal and model the importance of integrity and ethical conduct, and do not monitor for or use rewards or sanctioning to stimulate such conduct.

As noted, high-Mach employees are self-centered and goal-driven, and they are thus likely to be more sensitive than low-Machs to messages about what type of behavior is likely to result in the highest rewards for them and will adapt their own behavior accordingly. For example, Wilson et al. (1996, p. 287) describe “Machiavellianism as a kind of master strategy that includes both cooperative and defecting substrategies, plus a system of rules for when to use them.” Similarly, Kessler et al. (2010) note that high-Machs can use manipulation and deceit but can also be genuinely accommodating and respectful, depending on what seems most advantageous for achieving their goals in a given situation. While low-Machs may generally show more ethical behavior than high-Machs, high-Machs may be more sensitive to cues from the environment about which behaviors are rewarded.

High-Machs have a strong preference for money and power (Stewart and Stewart, 2006; Sakalaki et al., 2007) suggesting that they strongly value the extrinsic motivational aspects of their work (e.g., promotions, status, power, and money). We therefore expect that followers will show increased ethical forms of behavior under ethical leaders given that this behavior is clearly expected, monitored for, and rewarded by the leader, and that this positive relationship will even be stronger for high-Machs than for low-Machs due to high-Machs’ strong goal orientation and their sensitivity to rewards (Jones and Paulhus, 2009; Kessler et al., 2010). Also, ethical leaders’ own ethical behavior sends a signal to employees that such behavior will facilitate achieving a leadership position in the organization, encouraging high-Machs who strongly value positions of status and power to engage in vicarious learning and copy such ethical behavior. In contrast, low ethical leaders do not expect or monitor for ethical behavior and may send the signal that “anything goes.” Under such leaders, we expect that high-Machs do not show increased ethical behaviors and rather engage more in unethical means to reach their goals, including particularly deception and manipulation.

Scholars have argued that a communal and people orientation (showing respect, supporting and helping others) is an essential part of ethical leadership (e.g., Treviño et al., 2003; Kalshoven et al., 2011b; see also Den Hartog, 2015). This implies ethical leaders will emphasize the importance of and reward showing affiliative behavior. High-Machs are more sensitive to such rewards than low-Machs and are therefore likely to show increased affiliative behavior only when ethical leadership is high, not under low ethical leadership as such leaders do not emphasize the importance nor reward employees for supporting and helping colleagues, and helping others is not something that high-Machs would typically do if they did not explicitly expect to be rewarded for it (Dahling et al., 2009). We therefore hypothesize the following.

Hypothesis 1. Machiavellianism and ethical leadership will have interactive effects on affiliative OCB, such that the relationship between Machiavellianism and affiliative OCB will be more positive under highly ethical leaders than under low ethical leadership.

While generally high-Machs will show more unethical behavior than low-Machs, unethical behaviors by high-Mach employees should strongly decrease under ethical leaders. These leaders monitor follower behaviors on an ethical dimension, communicate clearly that unethical behaviors are not acceptable, and punish such behaviors when detected. This active monitoring decreases high-Machs’ room to maneuver and signals that unethical behavior is likely not to lead to reward but to punishment. As noted, high-Machs adapt their behavior to the situation and do not always engage in unethical behaviors; in particular, they do not show manipulation and deception if it is not advantageous or might even be detrimental for achieving their goals (Wilson et al., 1996; Kessler et al., 2010). Here, we investigate two specific types of manipulation and deception behavior, namely, knowledge hiding (e.g., Webster et al., 2008; Connelly et al., 2012) and (emotional) manipulation (Austin et al., 2007). Both of these behaviors can be labeled unethical behaviors. In this respect, Gini (1998) argues that, in order to act ethically, individuals need to consider and respect the interests and rights of all affected parties in their behaviors. Yet, when hiding knowledge from others, the knowledge hider accepts that the interests of others might be harmed due to a lack of information. Emotional manipulation refers to the instrumental use of reading and managing others’ emotions to suit one’s interests, even against the interests of others (Austin et al., 2007). Such behaviors are in conflict with being a “moral person” who carefully considers the consequences of one’s actions (cf. Treviño et al., 2000). Both knowledge hiding and emotional manipulation thus refer to behaviors that ignore and neglect others’ needs or interests and may even go against those needs in order to maximize satisfaction of one’s own (or one’s own group’s) interests and can thus be considered as unethical (see Den Hartog, 2015).

Knowledge hiding refers to employees’ efforts to withhold or conceal knowledge from colleagues rather than share it, even if that knowledge is useful for or needed by them (e.g., Connelly et al., 2012; Connelly and Zweig, 2015). Knowledge hiding is hence the opposite of sharing knowledge with and helping colleagues and forms the antisocial, unethical counterpart of pro-social affiliative OCB as it refers to an active and intentional attempt of employees to hide their knowledge from colleagues.

As noted, ethical leaders advocate communal and people-oriented behaviors (see Den Hartog, 2015), and knowledge and information sharing has been identified as part of ethical leadership (Kalshoven et al., 2011b). In line with this, followers of ethical leaders were found to show increased knowledge sharing (Ma et al., 2013). Thus, we argue that, under highly ethical leaders, high-Machs’ adaptivity and goal focus will not only lead to increased affiliative OCB but also to decreased knowledge hiding activities compared to low-Machs who are more likely to already be willing to share knowledge regardless of their leader’s behavior, as high-Machs likely perceive that under ethical leaders, who monitor them, knowledge hiding will be easily discovered and is detrimental for their career (Connelly et al., 2012). In contrast, leaders low on ethical leadership do not emphasize and monitor employee behavior on a moral dimension and thus are more likely to give the signal to high-Machs that they do not need to pay attention to ethical behaviors but can cut corners and deceive others without being punished. Given high-Machs predisposition to fall back on unethical behaviors (Jones and Paulhus, 2009), we thus expect their knowledge hiding to increase when ethical leadership is low compared to when it is high. We therefore hypothesize the following.

Hypothesis 2. Machiavellianism and ethical leadership will have interactive effects on knowledge hiding, such that the relationship between Machiavellianism and knowledge hiding will be less positive under highly ethical leaders than under low ethical leadership.

While knowledge hiding refers to an unethical behavior targeting specifically colleagues (Connelly et al., 2012), we argue that high-Machs’ tendencies to engage in unethical behaviors will generalize and also show in other social contexts (Christie and Geis, 1970; Jones and Paulhus, 2009). We therefore investigate a second unethical, antisocial behavior aimed at a different target, namely the use of manipulative behavior toward leaders. The use of manipulation is one of the defining characteristics of Machiavellianism (Christie and Geis, 1970) and an important part of measures of Machiavellianism (cf. Christie and Geis, 1970; Dahling et al., 2009; Kessler et al., 2010). Emotional manipulation is defined as manipulating others’ emotions within a self-serving framework (e.g., Grieve and Mahar, 2010) and has been positively linked to both Machiavellianism (Austin et al., 2007) and psychopathy (Grieve and Mahar, 2010), potentially because it is an effective but more covert type of manipulation compared to other manipulative behaviors (e.g., lying, providing false information). Due to this reduced risk of discovery and the power differential between leaders and followers, emotional manipulation seems a type of manipulation particularly suitable to be used by followers with their leaders.

Ethical leaders emphasize fairness, are trustworthy and honest, advocate integrity, and communicate the importance of such behaviors to employees (Brown et al., 2005; Kalshoven et al., 2011b). Ethical leaders thus do not use manipulation themselves and clearly signal to employees that manipulative behavior is not acceptable and will lead to negative consequences (see Den Hartog, 2015). As noted above, we argue that high-Mach employees are particularly sensitive to their leaders’ signals and expectations about desirable behaviors (e.g., Kessler et al., 2010) and will therefore avoid (or at least reduce) the use of manipulative behaviors under highly ethical leaders. Leaders low on ethical leadership, in contrast, do not discuss or model ethical behavior nor do they monitor or punish (un)ethical employee behaviors, and high-Machs should therefore more freely engage in emotional manipulation under such leaders. We therefore expect the following.

Hypothesis 3. Machiavellianism and ethical leadership will have interactive effects on emotional manipulation, such that the relationship between Machiavellianism and emotional manipulation will be less positive under highly ethical leaders than under low ethical leadership.

## Materials and Methods

### Procedure and Sample

We tested the three moderation hypotheses presented above in a multi-source survey study among 159 unique employee-supervisor dyads in Netherlands. We used business school contacts to get access to organizations and asked these organizations whether they would be willing to participate in a study on leadership in organizations and its impact on employees. The organization had to provide contact information of one of their employees and his/her supervisor. We then sent employee and supervisor a paper-and-pencil version of the survey by email accompanied by a letter explaining the purpose and purely academic nature of the study and the voluntary and confidential nature of participation. Respondents did not receive anything in return for participating in the study. After having read this information, respondents filled in the survey. The study was carried out in accordance with the recommendations of the Economics and Business Ethics Committee, University of Amsterdam, who approved the protocol for the study (request nr 20171124121141). In total, we sent out 240 employee–supervisor surveys, and we received 159 employee–supervisor dyads back, resulting in a response rate of 66%. Surveys were administered in Dutch. All survey scales came from validated measures and were carefully translated and back-translated by native speakers, respecting the norms of the International Test Commission.

Respondents worked in a wide range of industries including health services, IT, architecture, account management, consultancy, education, and financial services. Of the participating employees, 37% were male and 63% female. The mean age of the employees was 34.98 years (*SD* = 13.36), and the average tenure at their current organization was 6.80 years (*SD* = 8.85). In total, 40% of the employees had attained a university (master’s) degree. Of the participating supervisors, 57% were male and 43% female. The mean age of the supervisors was 42.23 years (*SD* = 12.15); their mean organizational tenure was 10.27 years (*SD* = 9.18). Supervisors had worked with the participating employee together for 3.21 years on average (*SD* = 3.73); 45% of the supervisors held a university master’s degree.

### Measures

Employees rated their own degree of Machiavellianism, their supervisors’ ethical leader behaviors, and their own knowledge hiding behaviors toward their colleagues and emotional manipulation toward their supervisor. Supervisors rated their employees’ affiliative OCB. All responses were measured on a seven-point Likert scale ranging from 1 (“completely disagree”) to 7 (“completely agree”).

Employee *Machiavellianism* was measured with eight items from the Mach-IV scale by Christie and Geis (1970) which is still the most widely used measure in studies on Machiavellianism. This Dutch eight-item short measure of Machiavellianism was used successfully in several recent studies in the Netherlands (e.g., Den Hartog and Belschak, 2012; Belschak et al., 2015, 2016). Sample items are “It is wise to flatter important people” and “Never tell anyone the real reason you did something unless it is useful to do so.” Cronbach’s alpha of the scale was 0.80.

Employees’ perception of their leaders’ *ethical leadership* was measured with the oft-used 10-item scale by Brown et al. (2005). This measure is well validated and was used in the Dutch context successfully before (e.g., Kalshoven et al., 2011a,b; Den Hartog and Belschak, 2012). Sample items are “My leader discusses business ethics or values with employees,” “sets an example of how to do things the right way in terms of ethics,” or “disciplines employees who violate ethical standards.” Cronbach’s alpha was 0.84.

Due to the conceptual overlap (e.g., caring about others, acting as role models for followers; see Brown and Treviño, 2006) and the substantial empirical correlations usually found between ethical and transformational leadership (e.g., Brown et al., 2005; Toor and Ofori, 2009; see Ng and Feldman, 2015), leadership scholars have emphasized the need to control for transformational leadership in studies regarding ethical leadership (see Den Hartog, 2015). We therefore also included transformational leadership in our survey and used the 11-item measure of the Dutch “Charismatic Leadership in Organizations (CLIO)” questionnaire to measure employees’ perception of their leaders’ *transformational leadership* (e.g., “My leader has a clear vision and an image of the future” and “stimulates subordinates to think independently”). This Dutch measure covers content similar to other measures of transformational leadership like the MLQ (e.g., Bass and Avolio, 1990; House, 1998). It is well validated and has been used in several leadership studies in the Netherlands before (e.g., De Hoogh et al., 2004, 2005; De Hoogh and Den Hartog, 2009). Cronbach’s alpha was 0.90.

*Affiliative OCB* was measured with seven items by MacKenzie et al. (1991). The items cover the helping and the courtesy dimension of this widely used multi-dimensional measure of OCB. Sample items are “This employee is always willing to help the people around him/her” and “considers the impact of his/her actions on others.” Cronbach’s alpha was 0.84.

The *knowledge hiding* scale is a relatively new measure which was first introduced by Connelly et al. (2012). We used seven items of this measure capturing all different strategies of knowledge hiding (playing dumb, evasive hiding, and rationalized hiding). Sample items read “When a colleague recently asked for information I agreed to help the colleague but provided different information than the requested one” and “I pretended that I did not know the information.” Cronbach’s alpha was 0.86.

The *emotional manipulation* measure was also relatively recently developed and introduced to the literature (see Kessler et al., 2010) and was taken from Austin et al. (2007). It consists of five items. Sample items are “I used my emotional skills to make my supervisor feel guilty” and “I made my supervisor feel uneasy.” Cronbach’s alpha was 0.92.

## Results

We conducted a confirmatory factor analysis to test the factor structure and the convergent and discriminatory validity of our scales. Statisticians have noted that a prerequisite for reliable results of a CFA is a satisfactory indicator to sample ratio (see, e.g., Bentler and Chou, 1987; Bentler, 1995). Due to the relatively high number of items compared to the sample size, we therefore used a parceling approach, as recommended (e.g., Bagozzi and Heatherton, 1994). For building the parcels, we followed a factorial algorithm by combining items into parcels according to the factor loadings of the items (e.g., Little et al., 2002; Rogers and Schmitt, 2004). To minimize loss of information, we only built parcels for the two longer and well-established leadership scales, and parcels consisted only of two items (and one parcel of three items in case of transformational leadership due to the uneven number of items). The CFA showed a satisfactory fit of the hypothesized six-factor structure (i.e., employee Mach, ethical leadership, transformational leadership, employee affiliative OCB, employee knowledge hiding, and employee emotional manipulation): χ^2^ (614) = 942.95 (*p* = 0.00); CFI = 0.90; IFI = 0.90; RMSEA = 0.06. Factor loadings were satisfactory ranging from 0.45 to 0.75 for employee Mach, from 0.53 to 0.82 for ethical leadership, from 0.73 to 0.89 for transformational leadership, from 0.56 to 0.72 for affiliative OCB, from 0.42 to 0.89 for knowledge hiding, and from 0.73 to 0.94 for emotional manipulation. Factor inter-correlations ranged from -0.33 (ethical leadership and emotional manipulation) to 0.78 (ethical leadership and transformational leadership).

While one of our dependent variables was rated by leaders (affiliative OCB), the other dependent variables (knowledge hiding and emotional manipulation) were measured as employee ratings and might hence be subject to common source bias. Such bias may inflate or deflate observed relationships between constructs (e.g., Podsakoff et al., 2003). To test for common method variance, we therefore included the same-source first-order common method factor to the CFA. This factor was defined as having as indicators all employee-rated items, and this controls for the portion of variance attributable to obtaining all measures from a single source (see Podsakoff et al., 2003). If common source variance played a role, factor loadings and/ or inter-correlations should differ substantially for CFAs including versus not including the common method factor. A comparison of the CFAs showed that factor loadings and factor inter-correlations were almost identical in both computations, thus suggesting that common source bias might not play a substantial role in our data.

The means, standard deviations, Cronbach’s alphas, and (Pearson) inter-correlations of the variables are presented in **Table 1**. Employee Mach was positively correlated with knowledge hiding (*r* = 0.42; *p* = 0.00) and emotional manipulation (*r* = 0.28; *p* = 0.00) and negatively correlated with ethical leader behavior (*r* = -0.20; *p* = 0.01). Consistent with earlier studies (e.g., Brown et al., 2005; Toor and Ofori, 2009), transformational and ethical leaderships were substantially correlated with each other (*r* = 0.65; *p* = 0.00), thus illustrating the need to simultaneously include both variables in subsequent analyses to be able to draw better conclusions about the unique effects of ethical leadership. Finally, employee affiliative OCB was significantly correlated with transformational leadership (*r* = 0.20; *p* = 0.01), but not correlated with ethical leadership, and knowledge hiding and emotional manipulation were both negatively correlated with ethical leadership (*r* = -0.23; *p* = 0.00; and *r* = -0.32; *p* = 0.00).

**Table 1:** Inter-correlations and descriptives of variables of interest.

	*M*	*SD*	1	2	3	4	5	6
1 Employee Mach	2.78	0.92	(0.80)					
2 Transformational leadership	5.42	0.85	-0.22**	(0.90)				
3 Ethical leadership	5.32	0.79	-0.20*	0.65**	(0.84)			
4 Affiliative OCB	5.56	0.75	-0.09	0.20*	0.13	(0.84)		
5 Knowledge hiding	1.80	0.91	0.42**	-0.16*	-0.23**	-0.13	(0.86)	
6 Emotional manipulation	1.75	1.09	0.28**	-0.15	-0.32**	-0.23**	0.50**	(0.92)

*N = 159. ^∗^p < 0.05. ^∗∗^p < 0.01.*

To test our hypotheses, we used the PROCESS macro (version 2.13.2; developed by Hayes, 2013) to conduct our analyses. More specifically, we regressed employee affiliative OCB, knowledge hiding, and emotional manipulation on employee Mach, ethical and transformational leadership, and the interaction term of employee Mach and ethical leadership. In the analyses, we used the PROCESS option to center the predictors around their respective means and based the interaction term (Mach x ethical leadership) on these mean-centered scores to ease interpretation. As research on OCB and on dark personality traits often includes demographics as control variables, we also added employee age and gender (1 = male, 2 = female; both measured as employee ratings) as well as leader age and gender (1 = male, 2 = female) and the length of the relationship between leader and employee (all three measured as leader ratings) as covariates. The results of the moderation analyses are presented in **Table 2**. Indeed most of the demographics were significantly related to our outcome variables.

**Table 2:** Results of the moderation analysis using the PROCESS macro.

	Affiliative OCB		Knowledge hiding		Emotional manipuation	
	*B* (*SE*)	*p*	*B* (*SE*)	*p*	*B* (SE)	*p*
Constant	4.51** (0.58)	0.00	2.12** (0.64)	0.00	1.88* (0.74)	0.01
Age employee	0.00 (0.01)	1.00	-0.01 (0.01)	0.13	-0.01* (0.01)	0.03
Gender employee	0.02 (0.13)	0.90	-0.28 (0.15)	0.06	-0.44** (0.17)	0.01
Age leader	-0.01 (0.01)	0.35	0.00 (0.01)	0.65	0.00 (0.01)	0.62
Gender leader	0.28* (0.12)	0.02	0.15 (0.14)	0.27	-0.05 (0.16)	0.75
Length of relationship	0.01 (0.02)	0.48	0.03 (0.02)	0.14	0.01 (0.02)	0.74
Employee Mach	-0.03 (0.07)	0.62	0.34** (0.08)	0.00	0.18* (0.09)	0.04
Ethical leadership	0.01 (0.10)	0.89	-0.19 (0.11)	0.09	-0.49** (0.12)	0.00
Ethical leadership × Mach	0.16* (0.08)	0.04	-0.19* (0.09)	0.03	-0.34** (0.10)	0.00
Transformational leadership	0.16 (0.09)	0.08	0.03 (0.10)	0.80	0.18 (0.12)	0.13
R^2^	0.10		0.27		0.28	

*N = 159. ^∗^p < 0.05. ^∗∗^p < 0.01.*

**FIGURE 1:**
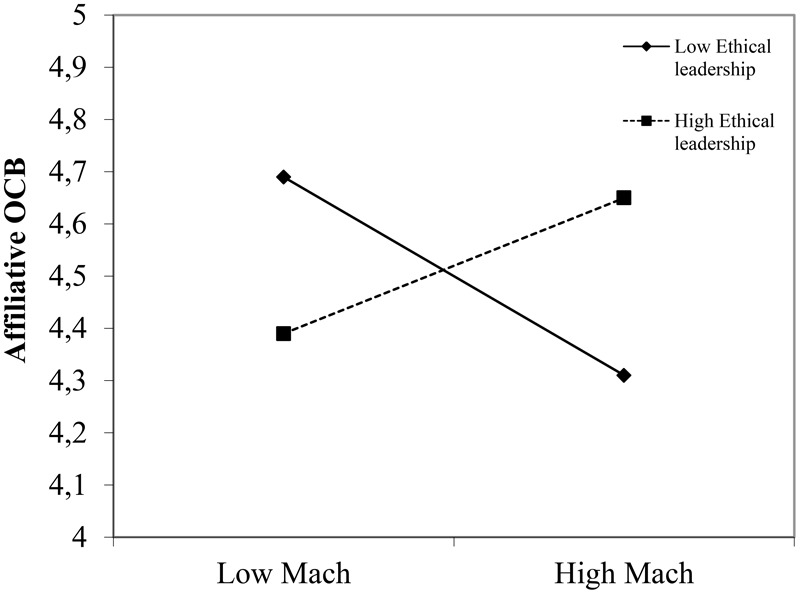
Interaction effect between ethical leadership and employee Machiavellianism for affiliative OCB.

**FIGURE 2:**
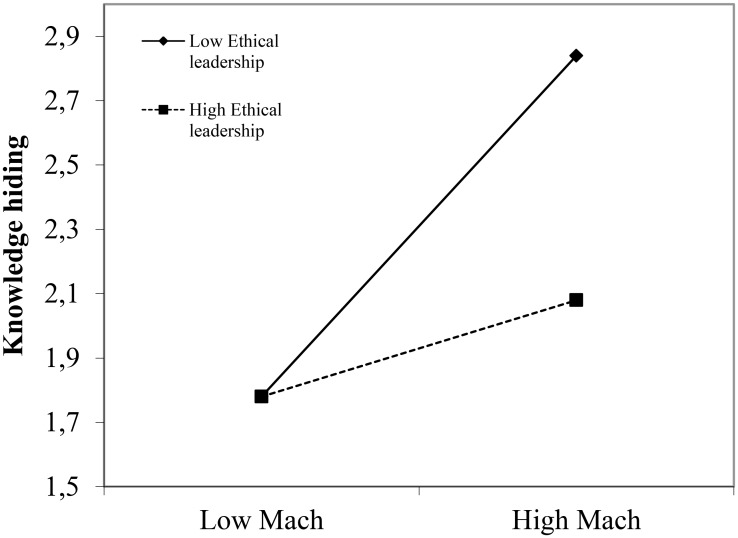
Interaction effect between ethical leadership and employee Machiavellianism for knowledge hiding.

**FIGURE 3:**
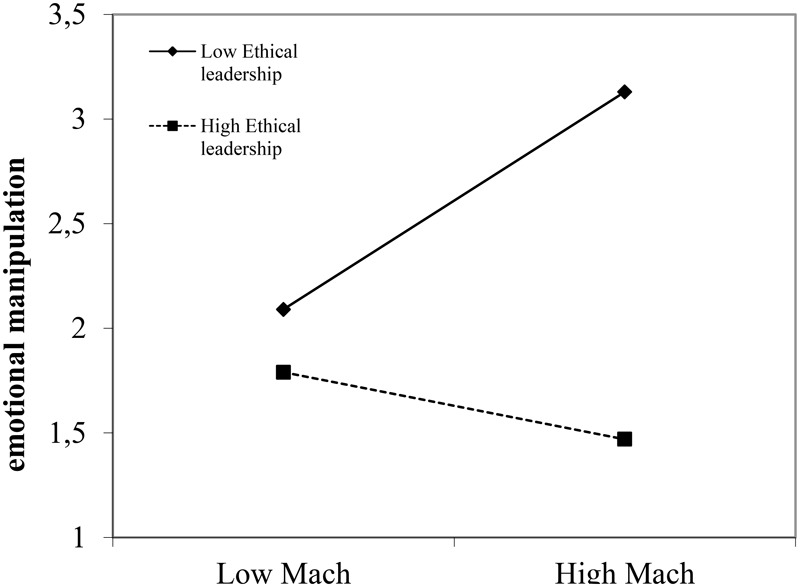
Interaction effect between ethical leadership and employee Machiavellianism for emotional manipulation.

Ethical leadership only had a significant main effect on emotional manipulation (*B* = -0.49, *p* = 0.00); the other main effects of ethical and transformational leadership were non-significant. More importantly though, and (mostly) in line with Hypotheses1–3, the main effects were qualified by significant interaction effects of employee Mach and ethical leadership for affiliative OCB (*B* = 0.16, *p* = 0.04), knowledge hiding (*B* = -0.19, *p* = 0.03), and emotional manipulation (*B* = -0.34, *p* = 0.00). To facilitate interpretation of these interaction effects, we plotted the relationship between employee Mach and the three outcome variables (affiliative OCB, knowledge hiding, and emotional manipulation) for high and low values of ethical leadership (**Figures 1–3**), while controlling for the effects of transformational leadership.

First, Mach is significantly and negatively related with affiliative OCB for low ethical leadership (*B* = -0.21, *p* = 0.05) but non-significantly for high ethical leadership (*B* = 0.13, *p* = 0.22, **Figure 1**). Next, the relationship between Mach and knowledge hiding is significant and positive for low ethical leadership (*B* = 0.53, *p* = 0.00) and non-significant for highly ethical leaders (*B* = 0.15, *p* = 0.21, **Figure 2**). Finally, the relationship between Mach and emotional manipulation is also significant and positive for low ethical leadership (*B* = 0.52, *p* = 0.00) and non-significant for highly ethical leaders (*B* = -0.16, *p* = 0.24, **Figure 3**).

## Discussion

High-Mach employees are a group of employees that is usually depicted as negative in the literature and sometimes even as dangerous for organizations (e.g., Dahling et al., 2009, 2012). Research has shown that high-Machs often make unethical choices and have the tendency to use manipulation and deception in social situations (e.g., Williams et al., 2010; Dahling et al., 2012). In line with this literature, we indeed found employee Mach to be significantly positively related to both hiding knowledge from colleagues and emotionally manipulating supervisors. Similarly, we replicated earlier findings that Mach is not significantly related to affiliative OCB (e.g., Dahling et al., 2009; Bagozzi et al., 2013). Yet, other authors found a negative link between Mach and OCB (e.g., Becker and O’Hair, 2007), suggesting that moderators might play a role and explain these inconsistent results in the literature. High-Machs might only help others if they expect to receive a reward in return for their help, for instance, using OCB as an impression management tactic to receive a more positive supervisor evaluation (Becker and O’Hair, 2007). We therefore investigated the interactive effects between employee Mach and supervisors’ leadership style on employee unethical behavior and OCB.

Belschak et al. (2015) have argued that leadership might offer the possibility to influence high-Mach employees’ behaviors in positive ways, and specifically they show that transformational leadership can increase high-Machs’ challenging OCB. However, they also caution this may not generalize to other outcomes. Building on this idea, we argued that high ethical leadership would reduce high-Machs’ unethical work behaviors and increase their motivation to show affiliative OCB, whereas low ethical leadership would have the opposite effect. Indeed, the relationship between Machiavellianism and affiliative OCB, knowledge hiding, and emotional manipulation was moderated by leaders’ ethical leadership. The findings of our study show that in particular under low ethical leadership high-Machs show undesirable reactions, while Machiavellianism was not significantly linked to affiliative OCB, knowledge hiding, and emotional manipulation when ethical leadership was high. Thus, low ethical leadership seems to trigger high-Machs to engage in more unethical behavior, whereas high ethical leadership suppresses the expression of such behavior by high-Mach followers, rather than high ethical leadership explicitly stimulating ethical behavior in high-Machs. By ignoring the ethical dimension in employee behaviors and not caring about or monitoring employees’ (un)ethical behavior, low ethical leaders seem to signal to their followers that it is acceptable to use unethical means and hence trigger undesirable behaviors particularly in high-Machs who have a predisposition to fall back on unethical behavior to achieve their ends.

Similarly, Greenbaum et al. (2017) found that high-Machs engage in unethical behavior under abusive supervisors and argue that abusive supervisors may provide cues that activate employees’ Mach trait, stimulating the expression of trait-consistent behavior. Our findings provide further support for the concept of Mach trait activation and for the notion that high-Mach employees can at least to some extent be managed as their behavior is linked to specific leadership styles (see Wilson et al., 1996; Belschak et al., 2015).

Somewhat surprisingly, we found that employees generally showed the highest affiliative OCB under low ethical leadership. A potential explanation of this unexpected finding is that colleagues may compensate for a lack of people-oriented leader behavior in a team. If followers are facing a lack of guidance, support, and help from their leader (i.e., low ethical leadership), they might look for and receive help from their colleagues who fall in and compensate for their leader’s deficiency. A similar compensatory model has been reported for perceived organizational support and perceived supervisor support (Maertz et al., 2007). Future research should further investigate this compensation mechanism in which followers step in for their leader and help each other where the leader fails to support them.

Literature on knowledge hiding has argued that such behavior harms the organization and thus, in turn, the knowledge hider him/herself (e.g., less money available for financial bonuses due to reduced work unit performance; e.g., Evans et al., 2015). If showing strategic and calculated behavior, high-Machs should thus avoid such behavior as they ultimately would also suffer themselves from its negative consequences. Yet, our findings show that knowledge hiding is strongly positively linked to Mach, despite of its potential for longer term detrimental effects. In this respect, the literature on Mach suggests that high-Machs might not adapt their behavior to potential longer term indirect effects (see Wilson et al., 1996). In game theoretical experiments, high-Machs aim for short-term profit maximization (e.g., Sakalaki et al., 2007) and easily change groups if needed (see Wilson et al., 1996); they thus seem more likely to strive for instant gratification than delayed rewards (see Christie and Geis, 1970). While knowledge hiding might harm the company in the long run, in the short run, it provides high-Machs with a source of power (cf. French and Raven, 1959) and status, hence giving them the opportunity to outperform others and achieve other external rewards (e.g., a bonus or promotion; Webster et al., 2008). Overall, the findings thus indicate that high-Mach individuals prioritize short-term profit maximization over long-term profit maximization, which would be of interest to test in future research.

Despite of the increased risk of targeting supervisors with unethical behavior, our results show that high-Machs not only engage in knowledge hiding toward colleagues but also in emotional manipulation toward their supervisors, in particular for supervisors low on ethical leadership. High-Machs’ tendency to use unethical behaviors when they have sufficient room to maneuver and the ethicality of their actions is not closely monitored thus seems to generalize to a broad range of manipulation and deception behaviors and to different targets. This result resonates with the results of an earlier study (Austin et al., 2007) which also found a positive link between emotional manipulation and Mach and extends it by introducing a contingency variable, (low) ethical leadership. While Mach was uncorrelated (Kessler et al., 2010) or even negatively linked with emotional intelligence in earlier studies (Austin et al., 2007), Bagozzi et al. (2013) found in fMRI studies evidence that high-Machs seem to use (non-conscious) emotional resonance processes which might allow them to “intuitively” feel and manipulate others’ emotions. Future research should further investigate the link between Mach and emotional manipulation and its underlying mechanisms.

A strength of our study is that we controlled for transformational leadership. Ethical leadership shows similarities with transformational leadership (e.g., the strong value orientation; see Brown and Treviño, 2006), and correlations between the two constructs are usually high (see Ng and Feldman, 2015). It is therefore important to control for transformational leadership in empirical studies on ethical leadership to be able to determine the variance explained uniquely by each construct (see Den Hartog, 2015).

### Practical Implications

Our findings offer several practical implications. First, high-Mach employees should be managed carefully. Our results show that high-Machs are sensitive to the behavior of their leaders and adapt their behaviors to leaders who emphasize and reward certain practices. Yet, our study also suggests that leadership effects seem to be limited to very specific employee behaviors. Leaders thus need to be clear and explicit to high-Mach employees about employee practices that are acceptable and those that are not. For instance, transformational leaders’ emphasis on change stimulates change-related behaviors like challenging OCB in high-Machs (Belschak et al., 2015), whereas ethical leaders’ focus on ethical behavior motivates them to avoid unethical work behaviors. In this respect, organizations are also well advised to introduce (ethical) organizational values and policies to communicate acceptable and desirable employee behaviors. Developing reward systems that clearly reward ethical behavior and punish unethical behavior could further help in establishing such norms and values.

Also, high-Machs seem to perceive a lack of specification of desirable behaviors as a signal that all means are acceptable to reach their goals and hence easily engage in unethical and other organizationally undesirable behaviors. High-Machs therefore form a group of employees that are particularly in need of guidance by leaders. While passive leadership generally comes with negative employee reactions in terms of increased incivility (Harold and Holtz, 2015), a lack of leadership seems to lead to even more pronounced effects for high-Machs who strongly fall back on unethical work behaviors that are particularly damaging to the organization.

Ethical leadership seems especially suitable to counter high-Machs’ tendency to engage in unethical work behaviors, and a lack of such leadership can be easily interpreted by high-Machs as a signal that “anything goes.” Fortunately, ethical leadership can be combined with other leadership styles like transformational or transactional leadership (see Den Hartog, 2015). It therefore seems good advice for leaders to always show ethical leader behaviors when high-Mach followers are part of their work unit. To suppress unethical behavior from these employees, organizations should therefore offer leadership training for leaders that particularly focuses on ethical leadership (emphasis on ethical behavior) and transactional leadership aspects (systematic use of monitoring, rewards, and punishments).

Finally, organizations might consider to include measures of Machiavellianism or (ethical) values in their personnel selection procedures. While measures of Mach are generally valid, it might be difficult though to measure high-Machs’ true personality during selection as this group of individuals is likely to manipulate their answers in socially desirable ways in situations in which they perceive the outcome may depend on a specific type of answer. In this sense, organizations might rather want to rely on long-term experiences of colleagues and supervisors to identify high-Machs and carefully consider this information in promotion decisions to avoid that high-Machs rise into higher management positions in the organizational hierarchy.

### Limitations

As most studies, this study also suffers from a number of limitations. First, we used a cross-sectional design and collected our data at one moment of time, and therefore we cannot make claims about the direction of causality. Experimental research is warranted to establish the direction of causality. For instance, work units that are characterized by highly unethical practices and an “all means are acceptable to meet one’s targets” employee attitude might be appealing to and attract in particular low ethical leaders. In this sense, it would also be interesting to investigate our topic from a longitudinal perspective and explore how processes unfold over time.

Second, we investigated only a limited range of employee behaviors. We have focused our study specifically on one ethical and two unethical behaviors, and our results suggest that high-Machs react clearly differently to leaders on these behaviors than on challenging OCB, as found in earlier studies. Thus, future research should further investigate and specify the different types of work behaviors that high-Machs adapt as a reaction to a specific leadership style. This would also be helpful for offering further advice to practitioners on how to manage high-Mach employees.

Also, our sample is not representative for the population of Dutch organizations, and there might be differences in ethical values across different industries that may have affected our findings. However, respondents in the study came from a broad range of different industries and organizations with no single industry being substantially more strongly represented than the others, which makes it unlikely that ethical values are systematically biased in any specific direction in our sample. Future research should consider and control for potential industry-related differences in ethical values and norms.

Finally, while we measured affiliative OCB as a supervisor rating and hence from a different source than the other variables in our study, employee Mach, leadership styles, knowledge hiding, and emotional manipulation were measured as employee ratings, which comes with the risk of common source variance. While our test for the effects of common source variance did not provide any evidence that common source bias may have affected our results, we cannot exclude this possibility. However, the main contribution of our study lies in the investigation of the interactive effects of employee Machiavellianism and leadership behaviors, and scholars have noted that analyses including interaction terms do not suffer from inflated interaction effects due to common method bias; rather, measurement error reduces the probability to find significant interactions (e.g., Busemeyer and Jones, 1983; Siemsen et al., 2010). Also, it is difficult to measure variables such as personality traits and covert, deceptive behaviors such as knowledge hiding and emotional manipulation through other ratings. In particular, there are currently no well-validated non-self-reported measures of Mach available. Future studies might collect leadership data from other sources though (e.g., colleague ratings) or develop other-rated measures of Mach thus including even more different data sources.

## Conclusion

This study investigated the link between leadership, employee Machiavellianism, and ethical (affiliative OCB) as well as unethical employee work behavior (knowledge hiding and emotional manipulation). We found that the relationship between Machiavellianism and these behaviors was strongly influenced by leaders’ ethical leadership style. Employee Machiavellianism came with reduced affiliative OCB and increased knowledge hiding and emotional manipulation, but only when ethical leadership was low. More research is warranted in the area of Machiavellians’ reactions to different leadership styles in order to help managing this group of organizational members.

## Ethics Statement

All subjects gave written informed consent in accordance with the Declaration of Helsinki. More specifically, at the beginning of the survey, respondents were informed about the content and purpose of the study; the academic nature of the study, i.e., the study was conducted by a university for research purposes; the voluntary nature of participation, i.e., participants did not receive anything in return for participation and participants were free to not respond to any question; and the fully anonymous nature of the study, i.e., it is impossible to identify either individual respondents or participating organizations.

## Author Contributions

FB, DDH, and ADH conceived and developed the project. FB and ADH coordinated and conducted data collection. All authors contributed to all parts of the manuscript, agreed to all aspects of the work, and approved the final version of the manuscript.

## Conflict of Interest Statement

The authors declare that the research was conducted in the absence of any commercial or financial relationships that could be construed as a potential conflict of interest.
